# Supplementary material for: Modeling glioblastoma heterogeneity as a dynamic network of cell states
Source: Mol Syst Biol. 2021 Sep 16;17(9):e10105. doi: 10.15252/msb.202010105 (PMC8444284; doi:10.15252/msb.202010105)
Supplement: Supplementary file 6 — Source Data for Figure 5 [file MSB-17-e10105-s004.zip › Figure5A_sourcedata/GSEA_3071/nosigdb_stateVI.GseaPreranked.1622795169056/RICHARDS_NOWAKOWSKI_SCIENCE_2017_OPC_UPREG.html]

Details for gene set RICHARDS\_NOWAKOWSKI\_SCIENCE\_2017\_OPC\_UPREG[GSEA]

|  || Dataset | state63071 |
| Phenotype | NoPhenotypeAvailable |
| Upregulated in class | na\_neg |
| GeneSet | RICHARDS\_NOWAKOWSKI\_SCIENCE\_2017\_OPC\_UPREG |
| Enrichment Score (ES) | -0.5584678 |
| Normalized Enrichment Score (NES) | -3.0034971 |
| Nominal p-value | 0.0 |
| FDR q-value | 0.0 |
| FWER p-Value | 0.0 |
Table: GSEA Results Summary

  

Fig 1: Enrichment plot: RICHARDS\_NOWAKOWSKI\_SCIENCE\_2017\_OPC\_UPREG      
 Profile of the Running ES Score & Positions of GeneSet Members on the Rank Ordered List

  

| PROBE | GENE SYMBOL | GENE\_TITLE | RANK IN GENE LIST | RANK METRIC SCORE | RUNNING ES | CORE ENRICHMENT || 1 | ALCAM |  |  | 7 | 1.185 | 0.0068 | No |
| 2 | FRMD6 |  |  | 8 | 1.177 | 0.0156 | No |
| 3 | NDUFAF2 |  |  | 211 | 0.595 | -0.0384 | No |
| 4 | EDIL3 |  |  | 219 | 0.589 | -0.0361 | No |
| 5 | NTNG1 |  |  | 357 | 0.509 | -0.0719 | No |
| 6 | NME1 |  |  | 405 | 0.487 | -0.0819 | No |
| 7 | HSPA5 |  |  | 414 | 0.484 | -0.0806 | No |
| 8 | SH3D19 |  |  | 482 | 0.460 | -0.0966 | No |
| 9 | PKP4 |  |  | 644 | 0.413 | -0.1401 | No |
| 10 | GALNT13 |  |  | 649 | 0.412 | -0.1382 | No |
| 11 | ARL4A |  |  | 704 | 0.395 | -0.1509 | No |
| 12 | OCIAD2 |  |  | 741 | 0.388 | -0.1584 | No |
| 13 | NT5E |  |  | 787 | 0.379 | -0.1686 | No |
| 14 | BCAT1 |  |  | 791 | 0.378 | -0.1666 | No |
| 15 | CPT1A |  |  | 832 | 0.371 | -0.1755 | No |
| 16 | DOCK10 |  |  | 858 | 0.367 | -0.1800 | No |
| 17 | ACP1 |  |  | 970 | 0.350 | -0.2095 | No |
| 18 | PDLIM5 |  |  | 1062 | 0.335 | -0.2333 | No |
| 19 | RPS27L |  |  | 1136 | 0.325 | -0.2520 | No |
| 20 | APCDD1 |  |  | 1157 | 0.323 | -0.2554 | No |
| 21 | SLC25A5 |  |  | 1163 | 0.321 | -0.2545 | No |
| 22 | GNG12 |  |  | 1178 | 0.319 | -0.2561 | No |
| 23 | EVI2A |  |  | 1221 | 0.313 | -0.2660 | No |
| 24 | TNS3 |  |  | 1232 | 0.311 | -0.2665 | No |
| 25 | NOC3L |  |  | 1310 | 0.302 | -0.2866 | No |
| 26 | KHDRBS3 |  |  | 1313 | 0.302 | -0.2849 | No |
| 27 | NTM |  |  | 1320 | 0.301 | -0.2844 | No |
| 28 | MRPL32 |  |  | 1342 | 0.299 | -0.2882 | No |
| 29 | EPB41L2 |  |  | 1343 | 0.299 | -0.2860 | No |
| 30 | UGDH |  |  | 1548 | 0.274 | -0.3430 | No |
| 31 | RALGPS2 |  |  | 1619 | 0.267 | -0.3613 | No |
| 32 | SFT2D1 |  |  | 1721 | 0.258 | -0.3886 | No |
| 33 | SDC3 |  |  | 1818 | -0.252 | -0.4145 | No |
| 34 | STXBP3 |  |  | 1827 | -0.253 | -0.4149 | No |
| 35 | LRP1B |  |  | 1882 | -0.261 | -0.4286 | No |
| 36 | PGRMC1 |  |  | 1889 | -0.262 | -0.4284 | No |
| 37 | COL11A1 |  |  | 1919 | -0.267 | -0.4348 | No |
| 38 | PHLPP1 |  |  | 1937 | -0.269 | -0.4377 | No |
| 39 | PCDH17 |  |  | 1955 | -0.271 | -0.4406 | No |
| 40 | OLIG2 |  |  | 1968 | -0.274 | -0.4421 | No |
| 41 | LRRTM3 |  |  | 2006 | -0.279 | -0.4507 | No |
| 42 | HAS2 |  |  | 2025 | -0.282 | -0.4538 | No |
| 43 | STK32A |  |  | 2031 | -0.283 | -0.4531 | No |
| 44 | TNRC6C |  |  | 2040 | -0.284 | -0.4533 | No |
| 45 | DOCK1 |  |  | 2063 | -0.289 | -0.4575 | No |
| 46 | SAMD8 |  |  | 2069 | -0.290 | -0.4568 | No |
| 47 | GATM |  |  | 2082 | -0.292 | -0.4581 | No |
| 48 | ALDH6A1 |  |  | 2091 | -0.294 | -0.4582 | No |
| 49 | FYN |  |  | 2118 | -0.301 | -0.4635 | No |
| 50 | SERPINE2 |  |  | 2128 | -0.302 | -0.4639 | No |
| 51 | TMEM132B |  |  | 2168 | -0.308 | -0.4729 | No |
| 52 | APBB2 |  |  | 2231 | -0.319 | -0.4884 | No |
| 53 | THY1 |  |  | 2265 | -0.326 | -0.4956 | No |
| 54 | ZNF462 |  |  | 2279 | -0.330 | -0.4969 | No |
| 55 | TNK2 |  |  | 2314 | -0.335 | -0.5042 | No |
| 56 | BMP7 |  |  | 2329 | -0.339 | -0.5057 | No |
| 57 | DENND5A |  |  | 2332 | -0.339 | -0.5038 | No |
| 58 | CHST3 |  |  | 2337 | -0.340 | -0.5024 | No |
| 59 | CNP |  |  | 2342 | -0.341 | -0.5010 | No |
| 60 | NAPEPLD |  |  | 2346 | -0.342 | -0.4993 | No |
| 61 | SEMA6D |  |  | 2377 | -0.349 | -0.5054 | No |
| 62 | BLM |  |  | 2409 | -0.355 | -0.5117 | No |
| 63 | ABAT |  |  | 2438 | -0.365 | -0.5171 | No |
| 64 | CSGALNACT1 |  |  | 2454 | -0.367 | -0.5187 | No |
| 65 | B2M |  |  | 2460 | -0.369 | -0.5174 | No |
| 66 | SEPT8 |  |  | 2472 | -0.370 | -0.5178 | No |
| 67 | ITGAV |  |  | 2476 | -0.371 | -0.5159 | No |
| 68 | PHYHIPL |  |  | 2512 | -0.381 | -0.5232 | No |
| 69 | GLCCI1 |  |  | 2521 | -0.383 | -0.5227 | No |
| 70 | IDH1 |  |  | 2526 | -0.384 | -0.5210 | No |
| 71 | SLITRK2 |  |  | 2541 | -0.387 | -0.5221 | No |
| 72 | ARHGAP42 |  |  | 2543 | -0.387 | -0.5196 | No |
| 73 | STRN |  |  | 2546 | -0.387 | -0.5172 | No |
| 74 | SPRY2 |  |  | 2569 | -0.391 | -0.5207 | No |
| 75 | ATCAY |  |  | 2585 | -0.396 | -0.5221 | No |
| 76 | ZMAT3 |  |  | 2590 | -0.399 | -0.5203 | No |
| 77 | KIAA0232 |  |  | 2603 | -0.402 | -0.5207 | No |
| 78 | DST |  |  | 2614 | -0.404 | -0.5206 | No |
| 79 | GLTP |  |  | 2653 | -0.412 | -0.5285 | No |
| 80 | SLC22A17 |  |  | 2662 | -0.414 | -0.5278 | No |
| 81 | DSEL |  |  | 2668 | -0.415 | -0.5261 | No |
| 82 | SEPT2 |  |  | 2703 | -0.426 | -0.5328 | No |
| 83 | CLDND1 |  |  | 2740 | -0.436 | -0.5399 | No |
| 84 | TMX3 |  |  | 2756 | -0.443 | -0.5410 | No |
| 85 | TENM1 |  |  | 2757 | -0.444 | -0.5377 | No |
| 86 | SLC2A13 |  |  | 2783 | -0.451 | -0.5415 | No |
| 87 | RAB31 |  |  | 2806 | -0.459 | -0.5445 | No |
| 88 | BAALC |  |  | 2819 | -0.462 | -0.5445 | No |
| 89 | QKI |  |  | 2844 | -0.474 | -0.5479 | No |
| 90 | PRKCA |  |  | 2847 | -0.474 | -0.5450 | No |
| 91 | TSPAN7 |  |  | 2848 | -0.475 | -0.5414 | No |
| 92 | CALCRL |  |  | 2853 | -0.478 | -0.5390 | No |
| 93 | CTTNBP2 |  |  | 2880 | -0.487 | -0.5429 | No |
| 94 | PON2 |  |  | 2884 | -0.490 | -0.5401 | No |
| 95 | SATB1 |  |  | 2899 | -0.494 | -0.5405 | No |
| 96 | CFLAR |  |  | 2902 | -0.495 | -0.5374 | No |
| 97 | ILDR2 |  |  | 2904 | -0.496 | -0.5340 | No |
| 98 | LAPTM4B |  |  | 2910 | -0.498 | -0.5317 | No |
| 99 | PDE4B |  |  | 2948 | -0.513 | -0.5386 | No |
| 100 | LAMA4 |  |  | 2959 | -0.520 | -0.5376 | No |
| 101 | SETD5 |  |  | 2993 | -0.537 | -0.5431 | No |
| 102 | SLC44A1 |  |  | 3013 | -0.546 | -0.5446 | No |
| 103 | CACNB2 |  |  | 3062 | -0.569 | -0.5542 | Yes |
| 104 | RFTN2 |  |  | 3065 | -0.570 | -0.5505 | Yes |
| 105 | FABP5 |  |  | 3079 | -0.577 | -0.5500 | Yes |
| 106 | SHC3 |  |  | 3093 | -0.581 | -0.5494 | Yes |
| 107 | PCDH9 |  |  | 3113 | -0.594 | -0.5505 | Yes |
| 108 | ERBB3 |  |  | 3116 | -0.596 | -0.5466 | Yes |
| 109 | TNFRSF21 |  |  | 3125 | -0.600 | -0.5445 | Yes |
| 110 | OLIG1 |  |  | 3126 | -0.601 | -0.5400 | Yes |
| 111 | SCD5 |  |  | 3135 | -0.611 | -0.5377 | Yes |
| 112 | SPATA6 |  |  | 3143 | -0.614 | -0.5352 | Yes |
| 113 | ITGB8 |  |  | 3148 | -0.616 | -0.5317 | Yes |
| 114 | GOLIM4 |  |  | 3165 | -0.634 | -0.5316 | Yes |
| 115 | ST3GAL5 |  |  | 3172 | -0.639 | -0.5286 | Yes |
| 116 | TM7SF3 |  |  | 3182 | -0.644 | -0.5264 | Yes |
| 117 | DNM3 |  |  | 3185 | -0.648 | -0.5222 | Yes |
| 118 | ITM2C |  |  | 3187 | -0.652 | -0.5176 | Yes |
| 119 | SKIL |  |  | 3188 | -0.652 | -0.5127 | Yes |
| 120 | AASS |  |  | 3191 | -0.657 | -0.5084 | Yes |
| 121 | PHLDB1 |  |  | 3194 | -0.657 | -0.5041 | Yes |
| 122 | GPR155 |  |  | 3206 | -0.675 | -0.5022 | Yes |
| 123 | MAGI1 |  |  | 3265 | -0.726 | -0.5136 | Yes |
| 124 | SOX2 |  |  | 3314 | -0.778 | -0.5217 | Yes |
| 125 | EMC10 |  |  | 3316 | -0.780 | -0.5162 | Yes |
| 126 | SALL3 |  |  | 3318 | -0.784 | -0.5106 | Yes |
| 127 | SCARB2 |  |  | 3320 | -0.789 | -0.5050 | Yes |
| 128 | SPRY4 |  |  | 3325 | -0.793 | -0.5002 | Yes |
| 129 | KCNQ1OT1 |  |  | 3332 | -0.796 | -0.4960 | Yes |
| 130 | DAG1 |  |  | 3336 | -0.803 | -0.4909 | Yes |
| 131 | TMEFF2 |  |  | 3344 | -0.808 | -0.4869 | Yes |
| 132 | DBI |  |  | 3349 | -0.812 | -0.4820 | Yes |
| 133 | GRIA2 |  |  | 3351 | -0.815 | -0.4762 | Yes |
| 134 | PCMTD2 |  |  | 3357 | -0.823 | -0.4715 | Yes |
| 135 | ZEB1 |  |  | 3363 | -0.827 | -0.4668 | Yes |
| 136 | NLGN1 |  |  | 3364 | -0.831 | -0.4606 | Yes |
| 137 | ETV1 |  |  | 3378 | -0.857 | -0.4580 | Yes |
| 138 | WSCD1 |  |  | 3379 | -0.860 | -0.4515 | Yes |
| 139 | ZNF385D |  |  | 3388 | -0.872 | -0.4473 | Yes |
| 140 | GNB4 |  |  | 3390 | -0.874 | -0.4411 | Yes |
| 141 | BCHE |  |  | 3405 | -0.909 | -0.4384 | Yes |
| 142 | CDH13 |  |  | 3410 | -0.913 | -0.4327 | Yes |
| 143 | NLGN4X |  |  | 3412 | -0.918 | -0.4262 | Yes |
| 144 | PLAT |  |  | 3416 | -0.927 | -0.4201 | Yes |
| 145 | LRRN1 |  |  | 3420 | -0.942 | -0.4140 | Yes |
| 146 | PROM1 |  |  | 3436 | -0.974 | -0.4110 | Yes |
| 147 | HIP1 |  |  | 3438 | -0.981 | -0.4040 | Yes |
| 148 | MAML2 |  |  | 3441 | -0.983 | -0.3972 | Yes |
| 149 | CD9 |  |  | 3445 | -0.995 | -0.3907 | Yes |
| 150 | CHD7 |  |  | 3454 | -1.017 | -0.3854 | Yes |
| 151 | ETV5 |  |  | 3466 | -1.043 | -0.3808 | Yes |
| 152 | PCDHGC3 |  |  | 3472 | -1.051 | -0.3744 | Yes |
| 153 | PLEKHH2 |  |  | 3478 | -1.060 | -0.3679 | Yes |
| 154 | SULF2 |  |  | 3486 | -1.086 | -0.3619 | Yes |
| 155 | TRIB2 |  |  | 3488 | -1.088 | -0.3540 | Yes |
| 156 | LCORL |  |  | 3490 | -1.089 | -0.3462 | Yes |
| 157 | LITAF |  |  | 3491 | -1.089 | -0.3381 | Yes |
| 158 | FGFBP3 |  |  | 3502 | -1.120 | -0.3326 | Yes |
| 159 | NOVA1 |  |  | 3510 | -1.148 | -0.3261 | Yes |
| 160 | MMP16 |  |  | 3511 | -1.150 | -0.3175 | Yes |
| 161 | DUSP6 |  |  | 3512 | -1.151 | -0.3089 | Yes |
| 162 | GPM6A |  |  | 3515 | -1.169 | -0.3007 | Yes |
| 163 | MYO10 |  |  | 3516 | -1.170 | -0.2920 | Yes |
| 164 | SOX6 |  |  | 3517 | -1.174 | -0.2833 | Yes |
| 165 | SEMA5A |  |  | 3526 | -1.210 | -0.2765 | Yes |
| 166 | LSAMP |  |  | 3537 | -1.257 | -0.2701 | Yes |
| 167 | ITM2B |  |  | 3540 | -1.269 | -0.2612 | Yes |
| 168 | IL1RAP |  |  | 3551 | -1.364 | -0.2539 | Yes |
| 169 | ITPR2 |  |  | 3556 | -1.388 | -0.2447 | Yes |
| 170 | CDCA7L |  |  | 3557 | -1.393 | -0.2343 | Yes |
| 171 | LIMA1 |  |  | 3564 | -1.432 | -0.2253 | Yes |
| 172 | SPRY1 |  |  | 3565 | -1.432 | -0.2146 | Yes |
| 173 | SLC35F1 |  |  | 3576 | -1.554 | -0.2059 | Yes |
| 174 | CADM4 |  |  | 3577 | -1.560 | -0.1943 | Yes |
| 175 | MAP3K1 |  |  | 3584 | -1.605 | -0.1841 | Yes |
| 176 | MAP2 |  |  | 3597 | -1.763 | -0.1744 | Yes |
| 177 | LRRN3 |  |  | 3606 | -1.871 | -0.1627 | Yes |
| 178 | SCRG1 |  |  | 3613 | -2.165 | -0.1483 | Yes |
| 179 | GPM6B |  |  | 3616 | -2.257 | -0.1320 | Yes |
| 180 | PMP2 |  |  | 3622 | -2.497 | -0.1148 | Yes |
| 181 | S100B |  |  | 3624 | -2.502 | -0.0965 | Yes |
| 182 | C1orf61 |  |  | 3626 | -2.616 | -0.0772 | Yes |
| 183 | PTN |  |  | 3632 | -2.826 | -0.0576 | Yes |
| 184 | PDGFRA |  |  | 3636 | -3.635 | -0.0313 | Yes |
| 185 | PTPRZ1 |  |  | 3637 | -4.237 | 0.0003 | Yes |
Table: GSEA details [plain text format]

  

Fig 2: RICHARDS\_NOWAKOWSKI\_SCIENCE\_2017\_OPC\_UPREG: Random ES distribution      
 Gene set null distribution of ES for **RICHARDS\_NOWAKOWSKI\_SCIENCE\_2017\_OPC\_UPREG**
